# Virome of respiratory secretion from children with unknown etiological acute respiratory disease revealed recombinant human parechovirus and other significant viruses

**DOI:** 10.1186/s12985-021-01586-0

**Published:** 2021-06-09

**Authors:** Ying Liu, Hao Wang, Jie Yang, Jian Zeng, Guang-Ming Sun

**Affiliations:** 1 Department of Clinical Laboratory, Xuzhou Central Hospital, 199 Jiefangnan Road, Xuzhou, 221009 Jiangsu China; 2 Department of Clinical Laboratory, The Affiliated Huai’an Hospital of Xuzhou Medical University, Huai’an, 223002 Jiangsu China; 3 School of Medicine, Jiangsu University, Zhenjiang, 212013 Jiangsu China

**Keywords:** Respiratory secretion, Viral metagenomics, Parechovirus, Anellovirus, Coxsackievirus

## Abstract

Using viral metagenomics, viral nucleic acid in 30 respiratory secretion samples collected from children with unknown etiological acute respiratory disease were investigated. Sequences showing similarity to human parainfluenza virus 1, anellovirus, bocavirus, coxsackievirus A4, human parechovirus (HPeV), and alphaflexivirus were recovered from these samples. Complete genomes of one anellovirus, one coxsackievirus A4, three parechoviruses were determined from these libraries. The anellovirus (MW267851) phylogenetically clustered with an unpublished anellovirus (MK212032) from respiratory sample of a Vietnamese patient, forming a separate branch neighboring to strains within the genus *Betatorquevirus*. The genome of coxsackievirus A4 (MW267852) shares the highest sequence identity of 96.4% to a coxsackievirus A4 (MN964079) which was identified in clinical samples from children with Hand, Foot, and Mouth Disease (HFMD). Two (MW267853 and MW267854) of the three parechoviruses belong to HPeV-1 and the other one (MW267855) belongs to HPeV-6. Recombination analysis indicated that an HPeV-1 (MW267854) identified in this study is a putative recombinant occurred between HPeV-1 and HPeV-3. Whether these viruses have association with specific respiratory disease calls for further investigation.

## Background

Globally, acute respiratory disease is the leading cause of morbidity and mortality among children under five years of age [1, 2] and viruses are the main causes of acute respiratory infections with the potential to cause pandemics. The ongoing coronavirus disease 2019 (COVID-19) pandemic emphasizes the need to actively study the virome of unknown etiological acute respiratory disease. Despite intensive laboratory investigations, the etiology of most acute respiratory infections is unknown, which makes clinical management difficult [3]. Viral metagenomics is a is an unbiased virus-detecting technique that is increasingly applied to non-specifically discover both already known and highly divergent viruses [4–6].

**Fig. 1:**
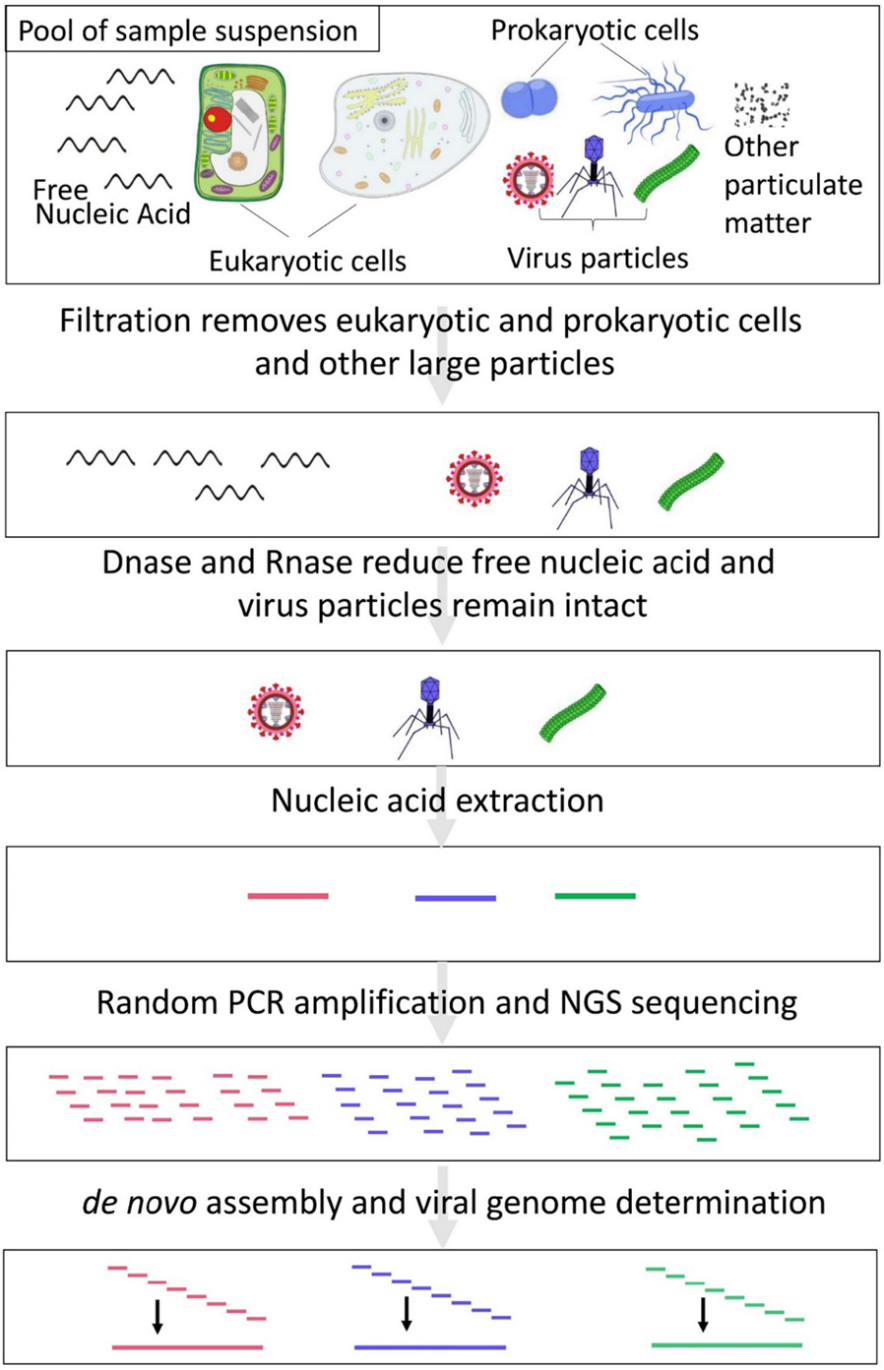
Schematic diagram outlines the typical viral metagenomic technique using filtration, nuclease, and extraction treatments to distinguish rare viral sequences from the abundant host cell and free nucleic acid

Here, using the viral metagenomic technique (Fig. 1), we investigated the virome of respiratory specimens collected from children presenting with acute respiratory infections and fully characterized the complete genomes of human parechovirus, anellovirus, and coxsackievirus identified from these samples.

## Findings

During 2019, 30 nasal-throat swab samples were collected from 30 children with age of < 5 years old with acute respiratory symptoms, whose pathogens (including virus and bacteria) were not identified at the Division of Clinical Microbiology of Xuzhou Center Hospital. Ethical Approval was given by the Ethics Committee of Xuzhou Central Hospital with the reference number as xzzx2018090. These samples were then subject to viral metagenomic analysis to investigate the virome. Briefly, tips of the swabs were immersed into 0.5 mL PBS and vigorously vortexed for 5 min and incubated for 30 min at 4 °C. The supernatants were then collected after centrifugation (10 min, 15,000×*g*) and randomly pooled into 3 sample pools each including 10 samples. Viral nucleic acid in the filtered Supernatant pool was then isolated using QIAamp MinElute Virus Spin Kit (Qiagen) according to the manufacturer's protocol. Three libraries were then constructed using Nextera XT DNA Sample Preparation Kit (Illumina) and sequenced using the MiSeqIllumina platform with 250 bases paired ends with dual barcoding for each pool. For bioinformatics analysis, paired-end reads of 250 bp generated by MiSeq were debarcoded using vendor software from Illumina. An in-house analysis pipeline running on a 32-nodes Linux cluster was used to process the data. Clonal reads were removed and low sequencing quality tails were trimmed using Phred quality score ten as the threshold. Adaptors were trimmed using the default parameters of VecScreen which is NCBI BLASTn with specialized parameters designed for adapter removal. The cleaned reads were de-novo assembled by SOAPdenovo2 version r240 using Kmer size 63 with default settings. The assembled contigs, along with singlets were aligned to an in-house viral proteome database using BLASTx with an E-value cutoff of < 10^−5^ [4, 7].

For phylogenetic analysis, virus nucleotide sequences (for coxsackievirus and HPeV) or deduced amino acid sequence of ORF1 (for anellovirus) were aligned using CLUSTAL W with the default settings [8]. Phylogenetic trees with 1,000 bootstrap resamples of the alignment data sets was generated using the Neighbour-Joining (N-J) method in MEGA7.0. Bootstrap values for each node were given [9]. For recombination analysis of HPeV, related genomes were retrieved from GenBank and subjected to multiple sequence alignment using MUSCLE in Mega 7.0. The recombination events were first assessed using RDP4.0 [10]. Finding putative recombination events where the genome identified here were involved, the related genomes were selected and realigned and recombination was confirmed by using similarity plot analysis in SimPlot software V. 3.5.1 [11]. Phylogenetic analysis was also performed based on recombinant regions to confirm the recombination event.

**Fig. 2:**
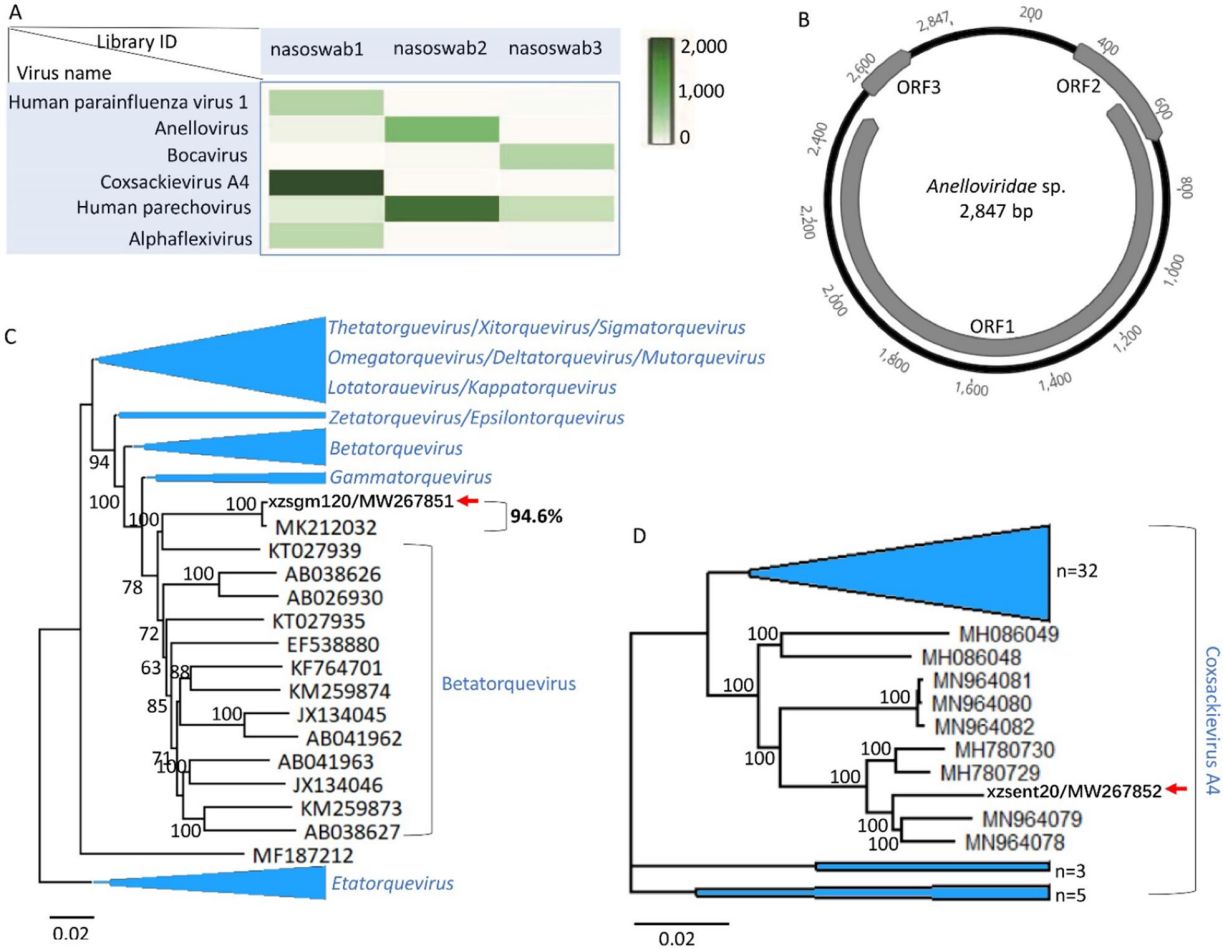
Sequence reads distribution of different viruses in respiratory secretion samples of children and phylogenies of anellovirus and coxsackievirus A4. **A** The counts of sequence reads aligning to different viruses are calculated and represented using a heat map. The color depth in each square represents the number of viral reads in each library. Names of virus detected in these libraries are labeled at left side and library IDs are labeled under the corresponding column. **B** Genome organization of the anellovirus (named xzsgm120 and GenBank no. MW267851) identified in this study. **C** Phylogenetic tree based on the amino acid sequence of the anellovirus identified in this study and other related anelloviruses. The anellovirus discovered in this study is indicated by a red arrow. **D** Phylogenetic tree based on the complete genome of the coxsackievirus A4 identified in this study and other related coxsackieviruses. The coxsackievirus A4 determined in this study is labeled by a red arrow

Next-generation sequencing (NGS) results indicated that the 3 libraries of 30 respiratory secretion samples generated a total of 1,278,128 sequence reads, where the 3 libraries contained 818,736, 330,598, and 128,794 sequence reads, respectively. BLASTx searching results indicated viral sequences showing similarity to human parainfluenza virus 1, anellovirus, bocavirus, coxsackievirus, human parechovirus (HPeV), and alphaflexivirus were detected in these samples (Fig. 2A), where sequence reads aligning to different viruses were counted and their log10 transformed values are represented using heat map (Fig. 2A). Sequence reads from the same species of virus were assembled within each barcode which generated five different complete virus genomes, including one anellovirus, one coxsackievirus and 3 HPeV genomes.

The circular genome of the anellovirus (named xzsgm120 from library nasoswab2) is 2847 nt long, of which the genome organization is consistent with those of other anelloviruses, including three ORFs (Fig. 2B). The ORF1, the largest ORF in this anellovirus, encodes a 664 amino acid long putative capsid protein. To determine the relationships of xzsgm120 to other anelloviruses, a phylogram was created based on the amino acid sequence encoded by ORF1. The Neighbour-Joining (N-J) tree (with 1,000 bootstrap resamples) based on the amino acid sequence of ORF1 indicated the anellovirus identified here closely clustered with an anellovirus (GenBank MK212032) from a respiratory sample of a Vietnamese patient according to the annotation in GenBank, forming a separate branch neighboring to those strains from the genus *Betatorquevirus* where they shared 94.6% sequence identity based on the complete genome sequence (Fig. 2C)*.*

One complete genome of coxsackievirus (xzsent20) was assembled and well grouped into the cluster of coxsackievirus, sharing the highest nucleotide sequence identity of 96.4% to a coxsackievirus A4 (CV-A4) (MN964079) which was identified from children with Hand, Foot, and Mouth Disease (HFMD) [12].

**Fig. 3:**
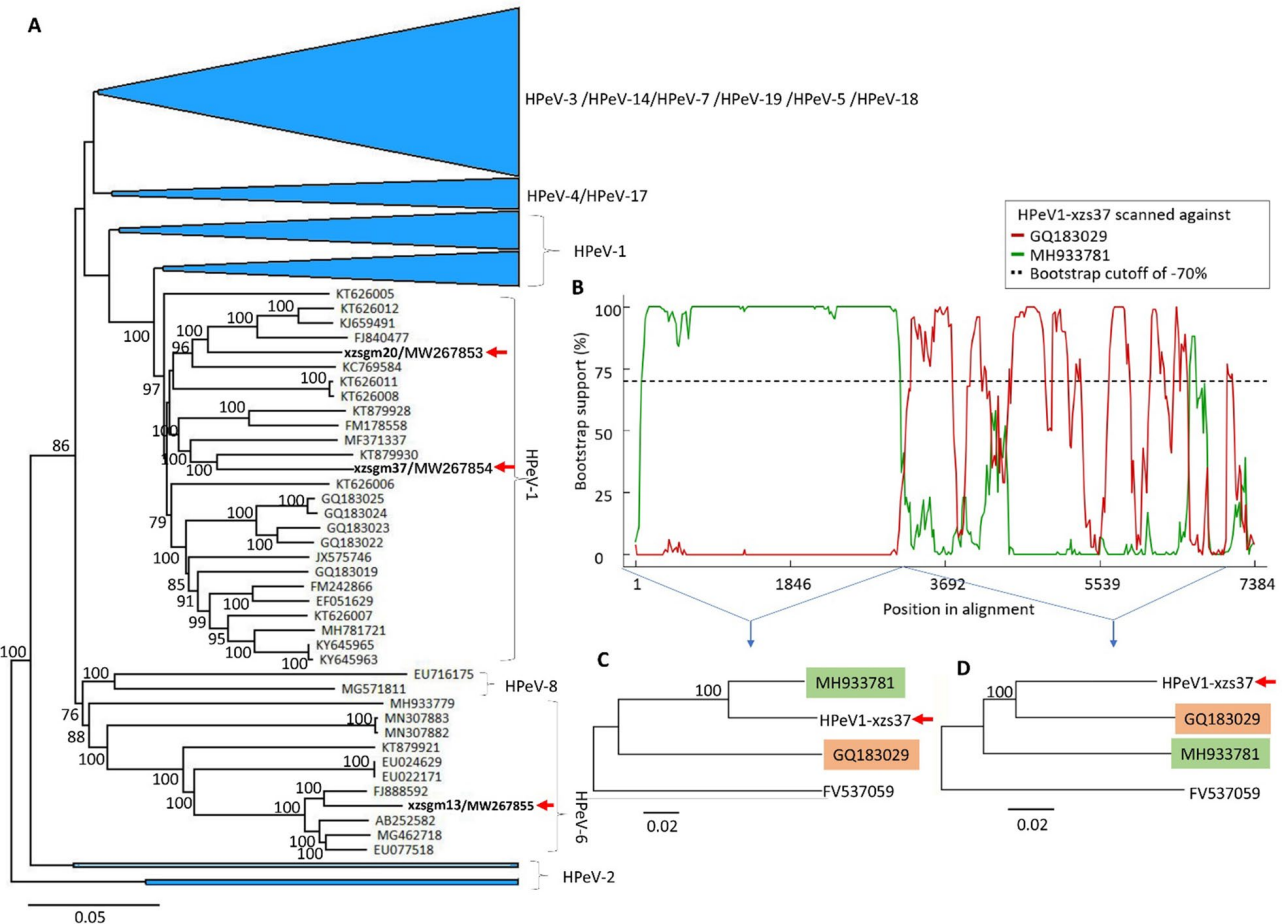
Phylogeny and recombination of HPeVs identified in respiratory secretion samples of children. **A** Phylogenetic tree based on the complete genome of the HPeVs identified in this study and other representative HPeV strains. **B** Bootscan evidence for the recombination of the HPeV-1 strain xzsgm37 (GenBank no. MW267854). **C** and **D** Phylogenetic trees respectively based on upstream and downstream sequences of the putative breakpoint in the recombination analysis. The HPeVs determined in this study are labeled by a red arrow

Three HPeV genomes were generated from the three libraries, respectively. Phylogenetic analysis based on the available complete genomes of 193 HPeVs in GenBank together with the 3 HPeV genomes determined here indicated two (xzsgm20 and xzsgm37) of HPeVs identified in this study were grouped into the cluster of HPeV-1 and the other one (xzsgm13) was closely related to HPeV-6, sharing 90.2–95.4% sequence identities based on the complete genome to their best BLASTn matches in GenBank (Fig. 3A). Recombination analysis using RDP4.0 software based on these 196 complete genomes suggested that genomic recombination occurred in one of the HPeV-1 (xzsgm37), where xzsgm37 seems to be a putative recombinant sequence produced by recombination event occurred between two parental lineages represented by an HPeV-1 strain (MH933781) and an HPeV-3 strain (GQ183029), respectively (Fig. 3B). The recombination event was further confirmed by phylogenetic trees based on upstream and downstream sequences of the putative breakpoint, respectively (Fig. 3C, D). To exclude the unnatural recombination due to assembly error during sequence assembly, primers covering the putative breakpoint were used to amplify the sequence fragment of xzsgm37 using cDNA products of library nasoswab01, and the amplified band was subjected to Sanger sequencing, which resulted sequence identical to the original genome sequence, suggesting this HPeV-1 strain is a natural recombinant.

## Discussion

Anellovirus is a common virus that can be detected in different tissues and organs of diverse species of animals [13–15]. An important feature of these viruses is the persistent infection in the host [16]. So far, there is no evidence showing a clear link between anellovirus and certain diseases. In the past decade, lots of studies have found that there is a positive correlation between anellovirus and the host immunosuppression. It has been suggested that the titer of anellovirus in plasma could be used as an indicator of immune recovery [14, 17, 18]. In the present study, anellovirus was detected in the respiratory tract of children with acute respiratory symptoms without known pathogens. Phylogenetic analysis revealed that this anellovirus showed a close relationship to another anellovirus which was also detected in respiratory samples, suggesting this anellovirus may have association with respiratory disease.

HPeV is a kind of nonenveloped virus with a single-strand positive RNA genome about 7.35 kb in length, including a single ORF [19]. HPeV is wildly prevalent in children population, although most of its infections are asymptomatic, it can be detected in samples from a variety of children's diseases, including skin rash, encephalitis, meningitis, sepsis, and even severe dilated cardiomyopathy [19–21]. HPeVs are now subdivided into > 16 different genotypes, among which some were associated with certain specific diseases [21, 22]. In this study, HPeVs were detected in the respiratory tract of children with acute respiratory disease, which included two different genotypes, HPeV-1, and HPeV-6. HPeV-1 is widely prevalent throughout the world and is often found in children with diarrhea and gastroenteritis [23]. HPeV-6 was first isolated from a cerebrospinal fluid specimen of a 1-year-old girl with Reye syndrome [24]. Detecting HPeV-6 in respiratory tract of children suggested HPeV-6 may transmit through respiratory tract and may cause central nervous system infection.

CV-A4 is classified as human enterovirus A (HEV-A) based on its serotype and is an etiological agent of HFMD. The virus can be detected in throat swabs from herpangina patients and can cause severe central nervous system symptoms [25, 26]. Our data indicated that a complete genome belonging to CV-A4 was present in the respiratory tracts of children with an acute respiratory symptom, suggesting CV-A4 may cause acute respiratory symptom and have potential of causing further infection in central nervous system.

## Conclusion

Taken together, we investigated the virome of 30 respiratory secretion samples collected from children with unknown etiological acute respiratory disease and fully characterized five genomes belonging to anellovirus, human parechovirus, and coxsackievirus. Whether these viruses have an association with acute respiratory disease needs further study with a larger sample size and healthy control cohort.

## Data Availability

The genome sequences of viruses described in detail were deposited in GenBank under the following accession numbers: MW267851- MW267855. The raw sequence reads from the metagenomic libraries were deposited in the Sequence Read Archive of GenBank database under the accession number: SRR12983513, SRR12983514, and SRR12983879.

## Abbreviations

BLAST: Basic local alignment search tool
NGS: Next-generation sequencing
HPeV: Human parechovirus
NCBI: National Center for Biotechnology Information
ORF: Open reading frame
CV-A4: Coxsackievirus A4
HFMD: Hand, foot, and mouth disease
